# Lack of a Synergistic Effect on Cardiometabolic and Redox Markers in a Dietary Supplementation with Anthocyanins and Xanthophylls in Postmenopausal Women

**DOI:** 10.3390/nu11071533

**Published:** 2019-07-05

**Authors:** Rocío Estévez-Santiago, José Manuel Silván, Cesar Abraham Can-Cauich, Ana Maria Veses, Inma Alvarez-Acero, Miguel Angel Martinez-Bartolome, Ricardo San-Román, Montaña Cámara, Begoña Olmedilla-Alonso, Sonia de Pascual-Teresa

**Affiliations:** 1 Department of Metabolism and Nutrition, Instituto de Ciencia y Tecnología de Alimentos y Nutrición (ICTAN-CSIC), C/José Antonio Novais, 10, 28040 Madrid, Spain; 2 Service Unit for Analytical, Instrumental and Microbiological Techniques (USTA) of Instituto de Ciencia y Tecnología de Alimentos y Nutrición (ICTAN-CSIC), C/José Antonio Novais, 10, 28040 Madrid, Spain; 3 Vascular and Interventional Radiology Department, Hospital 12 de Octubre, 28041 Madrid, Spain; 4 Departamento de Nutrición y Bromatología II, Facultad de Farmacia, Universidad Complutense de Madrid, Plaza de Ramón y Cajal s/n, 28040 Madrid, Spain

**Keywords:** anthocyanins, xanthophylls, lutein, zeaxanthin, metabolomics, inflammation, cardiometabolic

## Abstract

Fruits and vegetables are pivotal for a healthy diet due partly to their content in bioactive compounds. It is for this reason that we conducted a parallel study to unravel the possible effect on cardiometabolic parameters of the ingestion of anthocyanins, xanthophylls, or both groups of bioactives together in postmenopausal women. Seventy-two postmenopausal women were randomized into an 8-month parallel study: a group consuming 60 mg/day anthocyanins (Group A), a group consuming 6 mg lutein and 2 mg zeaxanthin per day (Group X), and a third group consuming a combination of anthocyanins and xanthophylls in the same amounts (Group A+X). Non-targeted metabolomic analysis was done in plasma samples at baseline and after the 8-month intervention by HPLC-QTOF-MS. Inflammatory, antioxidant, and cardiometabolic parameters were measured at the beginning of the study and after 4 and 8-months intervention. Compared with baseline values, none of the 8-month treatments significantly (*p* < 0.05) changed systolic or diastolic blood pressure (BP), plasma C-reactive protein, interleukin 6, vascular cell adhesion molecule-1, intercellular adhesion molecule-1, monocyte chemoattractant protein-1 or matrix metalloproteinases 2 and 9. Only plasma glucose levels were significantly decreased by treatment A+X after 8 months, and the plasma metabolomic profile was clearly affected by all three dietary supplementations after 8 months. In parallel, there was an increase, also for the three groups, in the plasma ferric reducing antioxidant power value that did not show any synergistic effect between the two groups of bioactives. Postmenopausal women could benefit from an increase in anthocyanins and xanthophylls intake, through the consumption of fruits and vegetables rich in these two types of compounds. Accordingly, plasma glucose and, above all, the reducing power in plasma, could be improved.

## 1. Introduction

It is generally accepted that the consumption of fruits and vegetables as part of a balanced diet has a positive effect on human health. Recently a meta-analysis of sixty-nine prospective studies has shown that vitamin C and carotenoids, as markers of fruits and vegetables consumption, are inversely associated with all causes of mortality, cancer, and cardiovascular disease [1]. Fruits and vegetables contribute a significant number of micronutrients and fiber to the diet that are already essential in establishing a balanced and healthy diet. However, in addition to these elements, they provide another series of components, so-called bioactives, which include flavonoids and carotenoids. Flavonoids and carotenoids have both been associated in different extensions and by different mechanisms, to protection against cardiometabolic disorders. For instance, anthocyanins have shown to reduce blood pressure [2]. This reduction can be explained by the anthocyanin effect at the nitric oxide biosynthetic pathway, interaction with the estrogen receptor or inhibitory effect on the angiotensin-converting enzyme (ACE) [3,4,5,6]. On the other hand, xanthophylls, such as lutein, have been shown to reduce C-reactive protein (CRP) plasma levels [7] which, in turn, has been shown to be one of the strongest independent predictors of future cardiovascular events [8]. Additionally, it has been proven that anthocyanin containing foods might have a multi-targeted effect in the so-called metabolic syndrome. On the one hand, anthocyanins exert their effect through the regulation of glycemic control [9], on the other, anthocyanins have also proved to affect the lipid metabolism at different levels, either in their biosynthesis or in their transport and distribution [10].

Many studies have failed to reproduce beneficial effects of isolated groups of bioactives alone. Therefore, it is hypothesized that the combination of different groups of antioxidants might have a beneficial effect on cardiometabolic health through the interaction with different metabolic and signaling pathways that are implicated in the onset of this group of conditions. Additionally, it has been largely proven that the cardiometabolic action of polyphenols in general and, anthocyanins in particular, [4] is estrogen mediated and that menstrual cycle effects might have an influence on the variability of the results found. Moreover, since there is an increase in the cardiovascular risk after menopause due to the lack of the protective effect of estrogenic hormones, it seemed to us that a population of postmenopausal women could be a good target group for our study. This is the reason we have conducted a parallel study to determine the effect of anthocyanin and xanthophylls on cardiometabolic health in postmenopausal women.

## 2. Material and Methods

### 2.1. Subjects and Study Design

Participants were selected from ninety women who were interested in participating in the study, after being contacted through advertisements in research centers, faculties, and several noticeboards in the Ciudad Universitaria Campus area. Inclusion criteria were age (50–70 years), amenorrhea (> 2y), BMI (25–33 kg/m^2^). Exclusion criteria were cholesterolemia (>240 mg/dL), use of drugs or foods to control cholesterol levels, use of restrictive diets, use of substitutive hormonal therapy, and chronic diseases that can affect any of the variables analyzed. Seventy-six women were enrolled and randomized into a parallel study of eight months duration and seventy-two completed the study. Participants were randomized in three groups: Group A anthocyanins (60 mg/d); Group X xanthophylls (6 mg lutein + 2 mg zeaxanthin/d); Group A+X anthocyanins (60 mg/d) + xanthophylls (6 mg lutein + 2 mg zeaxanthin/d). Supplements, in the form of soft capsules, were prepared by HC Clover Ps (Madrid, Spain) and consumed once per day.

Blood samples were collected at the start, and at 4 and 8 months after the intervention for the analysis of total polyphenols, lipid profile, and markers of inflammation and cardiovascular function. Metabolomics was performed in plasma samples at times 0 and after 8 months of supplementation using HPLC-Q-TOF LCMS. 

The study was conducted in accordance with the Declaration of Helsinki and the Spanish law14/2007 on biomedical research. The protocol was approved by the Ethics Committee for Clinical Research of the Hospital Universitario Puerta de Hierro-Majadahonda (Madrid, Spain) (Act 283, on December 17 2012) and the Bioethics Committee of the Spanish National Research Council (CSIC) (May 30 2014). Written informed consent was obtained from all subjects.

### 2.2. Preparation of Blood Samples

Peripheral blood was withdrawn by antecubital vein puncture from overnight fasted subjects into two types of tubes: one ethylenediamine tetraacetic acid (EDTA)-coated tube and one gel-containing tube for serum fraction separation (S-MONOVETTE^®^, Sarstedt AG & Co., Nümbrecht, Germany). Plasma or serum was generated by centrifugation at 1300 *g* for 15 min at 4 °C. Plasma aliquots from the EDTA tube were acidified with 100 µL cold 6% (w:v) metaphosphoric acid containing 1 mmol/L of EDTA and mixed for vitamin C analysis. EDTA plasma was also used for FRAP analysis, inflammatory cytokines (IL-6 and MCP-1), adhesion molecules (ICAM and VCAM), and matrix metaloproteinases (MMP2 and MMP9). Serum was used for glucose, insulin, cholesterol (total, HDL, and LDL cholesterol), triglycerides, iron, ferritin, complements C3 and C4, and high-sensitive C-reactive protein (hsCRP) analysis. Serum was also used for total polyphenol and metabolomic analysis. Briefly, two aliquots (1 mL) were acidified with 30 µL of 50% aqueous formic acid and samples were frozen at −80 °C until analysis.

### 2.3. Cytokines and Metaloproteinases Analysis

The levels of inflammatory cytokines, IL-6 and MCP-1, adhesion molecules, ICAM and VCAM, and matrix metaloproteinases, MMP2 and MMP9, in plasma were measured by using commercially available ELISA kits (RayBiotech, Peachtree Corners, GA, USA, for IL-6, ICAM-1. VCAM-1, and MMP9; Aviva Systems Biology, San Diego, CA, USA for MCP2 and Diaclone SAS, Besançon, France for MCP-1). Plasma was diluted with milliQ water as required (no dilution for IL-6 and MCP1; 1:100 for ICAM; 1:60 for VCAM; 1:1 for MMP9, and 1:2 for MMP2) for the quantification of the different molecules and the concentration in plasma was calculated with reference to the standard curves performed with the corresponding recombinant molecule.

### 2.4. Total Polyphenols and Ferric Reducing Antioxidant Power (FRAP) Analysis

Total polyphenols were analyzed by two methods, one the Folin–Ciocalteau micro-method as in Silvan et al. (2013) [11] and the Fast-Blue method [12] that was modified for its application to plasma samples. In both cases, plasma samples were first deproteinized by using isopropanol. Briefly, for the Fast-Blue method, 150 µL deproteinized plasma was placed in a 96-well plate well, and the reaction was started by adding 15 µL/well of 0.01% Fast Blue in water and 15 µL/well of 5% NaOH. After incubating the plate for 120 min at room temperature, the absorbance was recorded at 420 nm in a BioTek Synergy HT multi-mode microplate reader with BioTek’s Gen5TM software (BioTek Instruments Inc., Winooski, VT, USA). Data were expressed as gallic acid equivalents.

The ferric reducing ability of plasma (FRAP) assay was performed as previously described [13]. The FRAP method measures the capacity of, in this case, human plasma, to reduce the ferric ion into the ferrous ion at acidic conditions. Briefly, 10 µL plasma was placed in a 96-well microplate well (in triplicate), and 290 µL FRAP reagent was added. After 15 min incubation at 37 °C, the absorbance was measured at 593 nm. FRAP values were expressed as Trolox equivalents.

### 2.5. Untargeted Serum Metabolomics by HPLC-QTOF

Ten microliters of apigenin 0.1 mM was added to 1 mL serum as an internal standard. For the clean-up phase and purification of the biological matrix, SPE cartridges (Bond Elut C18, 500 mg 3 mL, Agilent) were used. After conditioning the cartridges with 2.5 mL 0.1% formic acid in methanol and 2.5 mL of 0.1% formic acid in water, the sample (1 mL) was loaded into the cartridge. Then, the cartridge was cleaned by elution with 3 mL aqueous 0.1% formic acid. Finally, the sample was eluted from the cartridge with 1 mL 0.1% formic acid in methanol. The elute was evaporated using a SpeedVac concentrator at room temperature. The residue was re-dissolved in 250 μL of formic acid 1 %/Methanol (50:50), filtered through a 0.45-μM filter, and analyzed by HPLC–QTOF. Analysis was performed by using an Agilent 1200 series liquid chromatographic system equipped with a quaternary pump, thermostatic auto-sampler, thermostated column compartment, and diode-array detector (DAD). High performance liquid chromatography coupled to quadrupole time-of-flight mass analyzer (HPLC-QTOF) with an electrospray ionization source (ESI) Jetstream technology was used. The spectra were recorded in negative and positive ion mode, and the MS detector was programmed to perform a consecutive scan series: extended dynamic range, low 1700 m/z. A 10 µL aliquot of sample was injected and analyzed in a Phenomenex Luna C18 column (150 × 4.6 mm, 3 µm). Two solvents were used during the analysis. Solvent A was composed of 0.1% formic acid in water, and solvent B was 0.1% formic acid in acetonitrile. The following gradient elution was used at a constant flow rate of 0.5 mL/min: (%B: 0 min 10%, 30 min 30%, 35 min 35%, 40 min 45%, 50 min 10%). Metabolomic analysis was done in the negative mode by using Masshunter Qualitative Analysis (B.07.00), Profinder (B.06.00), and Mass Profiler Professional (13.01.1) software (Agilent Technologies, Santa Clara, CA, USA). First, an ion extraction project of all the samples was recursively created by using Profinder. The extraction of ions from each sample was combined with the ion list of the rest in a single list after being aligned by retention time and exact mass. This new unique ion list was used by a recursive process for a specific search of all the ions in that list in each of the samples. With Massprofiler, a statistical analysis was first performed that resulted in a list of significant ions with *p* < 0.05. From this list, a PCA multivariate statistical study was performed to see the separation of the samples in the space of three coordinates as well as to know the intensity that each ion contributes to that separation by its value within the component to which they belong. The study was done by grouping all the samples at time 0 and 8 months and also by groups of treatment: A, X, and A+X. For metabolites identification different free accessible databases were used, including Human Metabolome Database, [14] ChemSpider (Royal Society of Chemistry), and Lipid Maps at https://www.lipidmaps.org, all of them between August 2018 and April 2019. HPLC–QTOF results in the positive mode were used to identify or confirm the identity of unknown compounds.

### 2.6. Statistics

Descriptive statistics (mean, standard deviation, 95% CI, and median) were calculated for all the analytes. The results are expressed as the mean ± standard deviation. First, data normality was confirmed for every variable. Statistical comparisons and differences according to treatment were assessed using one-way ANOVA and the post-hoc Bonferroni test. Relationships among variables in serum were established using Pearson’s correlation coefficient. Statistical significance was set at *p* < 0.05 or < 0.01 depending on the case and analysis were performed with IBM SPSS Statistics 22.0 (IBM Corp., Armonk, NY, USA)

## 3. Results

### 3.1. Study Outcomes: Biochemical and Cardiometabolomic Parameters

From the women that were contacted, 90 were initially appointed for a first screening date in which a first biochemical analysis was done. From them four were excluded because of having a high blood pressure and receiving treatment, two were medicated with drugs against hypercholesterolemia, one of them was undergoing hormone replacement therapy, two did not meet the requirement to have amenorrhea for two years, three did not meet the criteria of BMI and two decided not to participate.

From the 76 women who began the intervention, one subject withdrew because of a prescription for hypercholesterolemia, another was diagnosed with breast cancer, the third one did not tolerate the intervention, and the last one withdrew with no explanation. Finally, 72 people remained at the end of the intervention. There were no significant differences in baseline characteristics between the groups (Table 1).

No basal differences were observed in blood biochemical markers or systolic and diastolic blood pressure between groups. It has been already reported elsewhere [15] that there were no differences in the volunteer’s intake of macronutrient or micronutrients throughout the study time. However, there was a significant increase in the intake of lutein and zeaxanthin in groups A and X from time 0 to time 8 and a significant decrease in anthocyanins intake in all groups between time 0 and 4 months. 

Total polyphenols were analyzed by two methods, the Folin–Ciocalteau and the Fast-Blue method. We decided to include results from these two methods since they might have a different use. Total polyphenols by the Folin-Ciocalteau method is broadly used and, even showing some flaws, it is generally accepted and allows comparison with previous results. On the other hand, the Fast-blue method is less extended but was selected for being more specific for small phenolic molecules, such as gallic acid or other breakdown polyphenol derivatives [12]. 

We failed to find an increase in total polyphenols measured by the Folin-Ciocalteau method in any of the groups (Table 2). However, total polyphenols (TP) determined by the Fast-Blue method showed a significant but moderate positive correlation with FRAP (r = 0.310; *p* < 0.01) for all plasma samples. A slight but significant positive correlation was also shown for vitamin C and FRAP (r = 0.139; *p* < 0.05) and for TP by the Fast-Blue method and Vitamin C (r = 0.169; *p* < 0.05). Additionally, when the results were analyzed by time point, there was a significant increase of about 19% in TP by Fast-Blue values for Group A after 4 months that was maintained throughout the 8 months of the study. Surprisingly there was also a significant increase of about 17% in TP by Fast-Blue values for Group X but not for Group A+X.

Vitamin C was only significantly increased by a 15% related to the baseline value after 8-months intervention in Group A. The antioxidant power in plasma measured by the FRAP method was increased in every group after 4 months and maintained throughout the total intervention time. This antioxidant effect was more evident, however, in the case of Group A after 4 (20% and *p* < 0.002) and 8 months (17% and *p* < 0.011) and in Group A+X after 4 months (14% and *p* < 0.005).

From all the cardiometabolic parameters analyzed in this study, there was only a marginally significant (*p* = 0.059) increase in MMP-9 for Group A after 8-months intervention and a non-significant (*p* = 0.089) trend, in the same sense, for Group A+X. Additionally, there were statistically significant lower glucose plasma levels for Group A+X after the 8-month intervention compared with Group X (*p* = 0.009) and a slight but significant decrease in C3 complement levels in Group A+X after the 8-month intervention compared with Group X (*p* = 0.045) (Table 3).

### 3.2. Metabolomic Analysis

**Figure 1:**
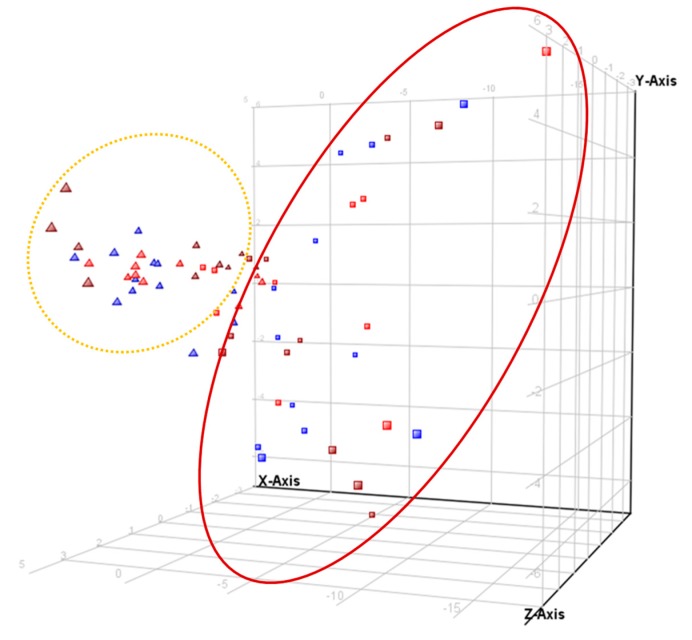
3D-PCA score plot for serum samples. Red (Group A), blue (Group X), and burgundy (Group A+X) triangles represent samples before intervention (time 0) and red, blue, and burgundy blocks after the 8-months intervention. X-axis represents component 1 (45.96%), Y-axis is component 2 (11.84%) and Z-axis, component 3 (6.78%).

By using Profinder, an ion extraction project of all the samples was recursively created. The extraction of ions from each sample was combined with the ion list of the rest in a single list after being aligned by retention time and exact mass. This new unique ion list was used by a recursive process for a specific search of all the ions in that list in each of the samples. A statistical analysis was performed that resulted in a list of significant ions with *p* < 0.05 by using Massprofiler. From this list, a PCA multivariate statistical study was performed to see the separation of the samples in the space of three coordinates as well as to know the intensity that each ion contributed to that separation by its value within the component to which they belong. This way, we found that there was a clear separation of the sample profiles by period, baseline, and 8-month samples (Figure 1).

Additionally, a list of ions that contributed to the differentiation of plasma samples for 0 and 8-month sampling points collected a total of 52 ions showing a *p* < 0.05. From them, some contributed almost equally to all post-treatment versus pre-treatment differences. However, other signals were associated with a given group. The study was done by grouping all the samples at time 0 and 8 months and also by groups of treatment: A, X, and A+X. A résumé of the ions and contribution is summarized in Table 4. Most of the ions that were regulated by period were common to the three treatments, meaning that there was actually an effect of the intervention during 8 months that was not really dependent on the type of bioactive. 

From those ions that contributed to a greater extension to the different situation of the samples in the PCA plot according to the treatment, we managed to preliminarily identify a number of them considering the mass score for a given molecular formula, the predicted fragmentation pattern, and with the help of different databases and scientific literature. When necessary, we also used data from the HPLC-QTOF analyses in the positive mode to confirm pre-identifications. The peak at 12.2 min with a mass of 173.104, corresponding to the molecular formula C8H15NO3 was tentatively identified as N-acetyleucine. This compound was downregulated by all treatments throughout the 8-months study time (Table 4).

The peak at 11.1 with mass 182.058 and molecular formula C9H10O4 was identified as 3,4-dimethoxybenzoic acid and was significantly upregulated in the combined diet Group A+X. The peak at 10.2 with mass 195.050 and molecular formula C9H9NO4 was upregulated in the A+X-group and was identified as 4-hydroxyhippuric acid. The peak at 21.6 with a molecular ion in the negative mode at 172.9918 and molecular formula C6H6O4S was tentatively identified as phenol sulfate and was significantly upregulated in the Group A+X. We were unable to pre-identify any of the other peaks in Table 4.

**Figure 2:**
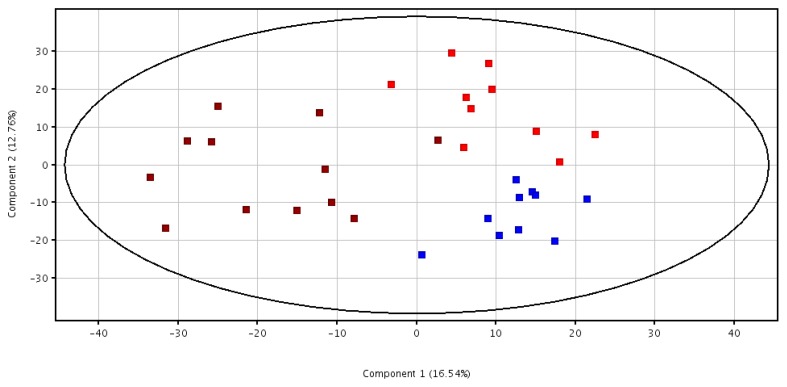
2D-PCA score plot for serum samples. Red (Group A), blue (Group X), and burgundy (Group A+X) after the 8-months intervention. X-axis represents component 1 (16.54%) and Y-axis is component 2 (12.76%).

Finally, we produced a list of ions that differentiated the three groups at the end of the study, after 8-months treatment. As it can be seen in Figure 2, the construction of the two axes plot does not fully explain the differences between the three treatments. However, a separation can be seen between Group A and Group X mostly explained by component 2 and between Groups A and X and Group A+X mostly explained by component 1.

A peak at 5.7 min with negative mass 338.0883 and predicted molecular formula C_15_H_17_NO_8_ was tentatively identified as 5-hydroxy-6-methoxyindole glucuronide. This peak was upregulated in Group A at time 8-months in comparison with Groups X (component 1 = 0.227 and component 2 = 0.197). The same occurred with a peak at 9.6 min with negative mass 191.0159 and predicted molecular formula C_6_H_8_O_7_ that was identified as citric acid (component 1 = −0.031 and component 2 = 0.197). Another peak was identified at a retention time of 47.4 min with a negative mass of 229.1445 and a molecular formula C_12_H_22_O_4_ as being dodecanedioic acid. This peak was present in all samples but upregulated in Group X. It helped to differentiate samples from this group from those in Group X and A+X (component 1 = 0.019 and component 2 = −0.012). 

## 4. Discussion

Many studies have proven the antioxidant and cardiometabolic active effect of fruits and vegetables as part of the diet. Fruits and vegetables are rich in fiber and antioxidant vitamins that per se can already cause such effects. In this work, we planned to prove that the group of polyphenols, Anthocyanins, and the carotenoids, Xanthophylls, have a role in the antioxidant and metabolic modulatory effect of fruits and vegetable consumption. Additionally, we wanted to prove that anthocyanins and xanthophylls, when consumed together, could, somehow, have a synergistic effect either by influencing each other’s absorption or metabolism or by influencing their effect by different or parallel mechanisms of action. 

Among all the analyzed inflammatory and cardiometabolic parameters only ferric reducing antioxidant activity (FRAP), glucose, and C3 complement were affected by dietary supplementation with either anthocyanins, xanthophylls, or the combination of both. In the case of the antioxidant power, as measured by the FRAP method, there was an increase in every group. However, as mentioned before, this effect was more evident in the case of Group A after 4 (20% and *p* < 0.002) and 8 months (17% and *p* < 0.011) and in Group A+X after 4 months (14% and *p* < 0.005). Nevertheless, the antioxidant effect did not show any synergistic or antagonistic effect between anthocyanins and xanthophylls. Moreover, our results show that anthocyanins on their own might have a sufficient effect on plasma redox state in our population and that the inclusion of an additional antioxidant, such as xanthophylls, to a potential formulation, does not really contribute much to the effect. Presently, it is generally accepted that reactive oxygen and nitrogen species contribute to the pathogenesis of cardiovascular diseases and inflammatory conditions. However, there is still no agreement by the scientific community regarding which should be the methodology used to determine the oxidative state in plasma. The FRAP method is an indirect method that measures the combined antioxidant effect of the non-enzymatic defenses [16]. It has been applied in different studies to human plasma showing, for instance, an inverse correlation with systolic and diastolic blood pressure [17] or its potential use as a prognostic biomarker in patients suffering from aortic stenosis [18].

Regarding plasma glucose levels for the three intervention groups, there was only a slight but significant decrease in plasma glucose levels for Group A+X after 8-months treatment. Previous work has shown controversial results regarding the effect of polyphenols on plasma glucose levels. Some authors have shown that even if moderate to low doses of cocoa or tea polyphenols might decrease glucose plasma levels; higher levels have no effect or even the opposite effect on plasma glucose levels [19]. Very interestingly, de Mello et al. (2017) [20] showed a negative correlation between hippuric acid (considered to be a metabolite produced by action of the colonic bacteria on anthocyanins) and the change in fasting plasma glucose levels, so the higher hippuric plasma levels are, the greater is the decrease in glucose plasma levels, suggesting that the regulatory effect of anthocyanins on plasma glucose levels might be mediated by the gut microflora.

Another parameter that was slightly affected by the A+X treatment was complement C3. Complement component 3 is part of the innate immunity of the immune response. A higher concentration of complement C3 in plasma is considered a diagnostic factor for different immune and cardiometabolic conditions. Complement C3 is also affected by levels of free fatty acids, glucose, and insulin and has been shown to be influenced by the postprandial state [21]. In our case, the decrease in complement C3 plasma levels for Group A+X was always within normal physiological levels and, if any, it could indicate an effect at the fatty acid or glucose metabolic level.

Total polyphenols measured by the Fast-Blue method in plasma was significantly increased after 4 months of intervention in Groups A (19.0%) and X (17.3%) and showed a positive correlation with FRAP antioxidant power. These results were unexpected in the case of Group X because it has been shown that the Fast-Blue method is more specific and shows fewer interferences than other methods for the quantitative determination of total polyphenols in plasma [22]. However, it could also be that there is a confounding factor introduced by the polyphenols present in the diet since we detected differences in the ingestion of anthocyanins and xanthophylls in the different treatment groups [15].

### Metabolomic Analysis

The analysis of plasma metabolomics showed the modulation of different ions. Some of the tentatively identified compounds were modulated in all three groups when comparing baseline samples with 8-months samples. This was the case for N-acetyleucine. N-acetyleucine is an endogenous metabolite of leucine, which is a protein precursor amino-acid but also has important regulatory functions, such as glucose metabolism regulation [23]. Additionally, N-acetylation of leucine might be mediated by gut microbiota, regulating the interplay between gut microbiota, amino-acid, and fatty acid metabolism and their implications in obesity and diabetes [24]. Our results show a decrease in N-acetyleucine plasma levels that is common for all three treatments after 8 months. This could indicate a decrease in leucine metabolism and thus, an increase in the availability of leucine for protein formation and regulatory functions.

3,4-Dimethoxybenzoic acid was also tentatively identified as being upregulated only in the A+X treated group. Different benzoic acid derivatives have been reported as biomarkers of quercetin (benzoic acid and 4-ethylbenzoic acid) or epigallocatechin gallate (1,3,5-trimethoxybenzene) dietary intake [25]. In our case, 3,4-dimethoxybenzoic acid could be formed by microbial metabolism of anthocyanins and its formation favored by xanthophylls co-administration. 3,4-Dimethoxybenzoic acid has shown anti-inflammatory activity in human gingival fibroblasts [26]. However, we did not find any effect of any of the treatments on any of the inflammatory markers analyzed.

4-Hydroxyhippuric acid has been identified as a metabolite in plasma and urine, after ingestion of an anthocyanin-rich berry extract [27,28] and in urine after a fruit and vegetable rich diet [29]. In our study, 4-Hydroxyhippuric acid was tentatively identified as part of the ions that were upregulated by the anthocyanin and xanthophylls combined diet (Group A+X). It might be that the co-ingestion of anthocyanins together with xanthophylls increases the production of this microbial end-product derived from anthocyanin metabolism by the intestinal microbiota.

There are very few references in the literature regarding 5-Hydroxy-6-methoxyindole glucuronide. However, it is part of the indole pathway that has been reported to be involved in inflammation defense and in melanogenesis. As a glucuronide is considered to be a waste product [14], which would imply the inhibition of the accumulation of indole itself in the system. It has been shown that Indole is produced by gut microbiota from tryptophan and that its augmentation leads to a decrease in motor activity and the accumulation of neurodepressant indole derivatives in the brain and eyes of indole treated rats [30]. Therefore, it could be expected that a decrease in the accumulation of indole and its oxidation products by the formation of more polar derivatives, likely to be eliminated, could have a beneficial effect at this level, inhibiting these neurodepressant effects.

Even if we showed some years ago that anthocyanins have an influence on human gut microbiota in vitro [31], recent studies contradict these results in vivo, showing a lack of effect on gut microbiota of a daily anthocyanin dose as high as 293 mg [32]. However, either by modulation of the microbiota or by influencing its metabolic capacity, the fact is that the regulation of the levels of metabolites derived from the action of such microbiota has been largely reported and agrees with our results [33,34,35].

In all, we need to take our results with caution due to the lack of placebo group and to the fact that there were no dietary recommendations or control, other than taking the product every day preferably in the morning. Nevertheless, we should consider that in our study, the doses of anthocyanins and xanthophylls, lutein and zeaxanthin, used were in the low range and could be considered as physiological and totally reachable within a healthy diet rich in fruits and vegetables. In spite of this, in our intervention, we observed a slight but significant change in the plasma metabolomic profile. Likewise, a certain reducing effect of plasma glucose levels has been shown, and a very important antioxidant effect has also been observed in all cases, whether anthocyanins or xanthophylls were administered alone or both groups of compounds were administered in combination.

## Figures and Tables

**Table 1:** General baseline characteristics of the subjects according to intervention group or total (mean ± SD).

Variable	Group A (*n* = 23)	Group X (*n* = 26)	Group A+X (*n* = 23)	Total (*n* = 72)
Age (years)	58 ± 6	60 ± 6	60 ± 5	59 ± 6
BMI (kg/m^2^)	24.9 ± 3.3	24.8 ± 2.8	24.5 ± 2.7	24.8 ± 2.9
Cholesterol (mmol/L)	5.41 ± 1.00	5.45 ± 0.69	5.75 ± 0.58	5.53 ± 0.78
HDL- cholesterol (mmol/L)	1.64 ± 0.33	1.72 ± 0.33	1.85 ± 0.35	1.73 ± 0.34
LDL-cholesterol (mmol/L)	3.77 ± 1.10	3.73 ± 0.77	3.91 ± 0.61	3.80 ± 0.84
TG (mmol/L)	1.04 ± 0.37	0.95 ± 0.38	0.92 ± 0.30	0.97 ± 0.35
Systolic blood pressure, (mm Hg)	118 ± 17	115 ± 14	121 ± 12	118 ± 15
Diastolic blood pressure, (mm Hg)	76 ± 11	74 ± 10	79 ± 9	76 ± 10
Glucose (mmol/L)	4.98 ± 0.47	5.13 ± 0.41	4.89 ± 0.38	5.01 ± 0.43

**Table 2:** Total polyphenols, vitamin C, and FRAP results for the three groups at the three time points (mean ± SD).

	Group A	Group X	Group A+X
	Mean ± SD		
Total Polyphenols (FB as g/L GAeq) – baseline	0.142 ± 0.028 ^a^	0.138 ± 0.029 ^a^	0.146 ± 0.018 ^a^
4 m	0.169 ± 0.026 ^b^ *	0.162 ± 0.032 ^b^	0.155 ± 0.036 ^a^
8 m	0.159 ± 0.029 ^ab^	0.153 ± 0.024 ^ab^	0.151 ± 0.034 ^a^
Total Polyphenols (F g/L GAeq) – baseline	0.249 ± 0.045 ^a^	0.272 ± 0.066 ^a^	0.247 ± 0.037 ^a^
4 m	0.217 ± 0.077 ^a^	0.221 ± 0.084 ^b^	0.224 ± 0.053 ^a^
8 m	0.209 ± 0.031 ^a^	0.237 ± 0.072 ^ab^	0.220 ± 0.060 ^a^
Vitamin C (µmol/L) – baseline	52.79 ± 13.40 ^a^	50.42 ± 8.4 ^a^	49.83 ± 12.47 ^a^
4 m	48.44 ± 16.12 ^a^	50.90 ± 13.31 ^a^	54.66 ± 19.14 ^a^
8 m	60.94 ± 14.90 ^b^	58.41 ± 17.83 ^a^	55.78 ± 19.15 ^a^
FRAP (Trolox eq) – baseline	588 ± 78 ^a^	568 ± 75 ^a^	569 ± 75 ^a^
4 m	704 ± 117 ^b^ *	677 ± 95 ^b^	650 ± 116 ^b^ *
8 m	687 ± 136 ^b^	661 ± 72 ^b^	642 ± 115 ^b^

Significance was set at *p* < 0.05, otherwise it is indicated by * *p* < 0.01. Different lower-case letters indicate significant differences between periods within a given treatment.

**Table 3:** Cardiometabolic and inflammation parameters for the three groups at the different time points (mean ± SD).

	Group A	Group X	Group A+X
	Mean ± SD		
SBP (mm Hg) – baseline	118 ± 17	115 ± 14	121 ± 12
4 m	118 ± 17	115 ± 17	118 ± 15
8 m	120 ± 16	113 ± 13	120 ± 12
DBP (mm Hg) – baseline	76 ± 11	74 ± 10	79 ± 9
4 m	75 ± 9	74 ± 10	78 ± 9
8 m	76 ± 11	74 ± 9	76 ± 10
Glucose (mmol/L) – baseline	4.98 ± 0.47	5.13 ± 0.41	4.88 ± 0.38
4 m	5.12 ± 0.50	5.12 ± 0.37	4.98 ± 0.37
8 m	5.12 ± 0.37	5.07 ± 0.56	4.75 ± 0.33 *
Insulin (pmol/L) – baseline	49.3 ± 21.5	50.7 ± 29.9	41.0 ± 14.0
4 m	50.7 ± 26.4	52.8 ± 27.1	47.2 ± 23.6
8 m	55.6 ± 23.6	50.7 ± 25.0	45.8 ± 22.2
C3 (g/L) – baseline	1.02 ± 0.02	1.06 ± 0.01	1.00 ± 0.01
4 m	1.09 ± 0.02	1.14 ± 0.02	1.04 ± 0.01
8 m	1.09 ± 0.02	1.06 ± 0.02	0.98 ± 0.01 *
C4 (g/L) – baseline	0.29 ± 0.01	0.29 ± 0.01	0.30 ± 0.01
4 m	0.31 ± 0.01	0.30 ± 0.01	0.29 ± 0.01
8 m	0.30 ± 0.01	0.29 ± 0.01	0.26 ± 0.01
CRP (mg/L) – baseline	1.23 ± 0.89	1.50 ± 1.78	1.38 ± 1.32
4 m	1.78 ± 1.90	1.77 ± 2.18	1.53 ± 1.99
8 m	1.26 ± 1.15	1.40 ± 1.23	1.22 ± 1.48
IL-6 (pg/mL) – baseline	0.8 ± 0.2	1.0 ± 0.6	1.1 ± 0.5
8 m	1.4 ± 1.9	1.1 ± 0.8	1.2 ± 0.9
ICAM-1 (ng/mL) – baseline	381 ± 208	344 ± 179	346 ± 192
8 m	351 ± 122	320 ± 132	345 ± 106
VCAM-1 (ng/mL) – baseline	185 ± 76	174 ± 62	152 ± 52
8 m	166 ± 54	160 ± 66	149 ± 83
MCP-1 (pg/mL) – baseline	126 ± 29	127 ± 30	137 ± 40
8 m	135 ± 65	137 ± 67	137 ± 48
MMP2 (µg/mL) – baseline	215 ± 63	257 ± 110	275 ± 161
8 m	222 ± 79	279 ± 115	275 ± 120
MMP9 (µg/mL) – baseline	93 ± 30	98 ± 42	85 ± 39
8 m	117 ± 43	104 ± 33	113 ± 38

Significance was set at *p* < 0.05. * difference between diets.

**Table 4:** Ions regulated from baseline to 8-months intervention in the three groups.

Theoretical Mass (*m/z*)	RT	*pA*	*pX*	*pA+X*	A	X	A+X
112.017	4.4		0.0251				
112.026	3.3			0.0044			
126.032	5.4	0.0206					
136.038	3.1			0.0394			
138.043	3.1	0.0271	0.0337				
166.050	3.0	0.0428		0.0479			
173.104	12.2	0.0024	0.0200	0.0000			
174.018	3.3		0.0473	0.0244			
174.008	21.6			0.0099			
182.058	11.1			0.0353			
187.120	18.7	0.0223	0.0066				
188.104	29.3		0.0087	0.0123			
195.050	10.2			0.0325			
201.136	25.9	0.0016	0.0055	0.0000			
215.152	33.0		0.0105				
219.057	5.4		0.0484				
236.934	2.1			0.0213			
244.071	3.3			0.0254			
306.059	4.4		0.0224				
308.924	5.1		0.0486				
318.154	2.7	0.0212					
340.234	37.7		0.0003	0.0073			
375.228	14.4			0.0024			
403.925	2.1	0.0272					
582.398	21.5	0.0382					
711.357	19.7		0.0373				
785.427	9.5	0.0493					

Green blocks indicate up-regulation with treatment, red blocks down-regulation.
